# Nicotinamide, a Poly [ADP-Ribose] Polymerase 1 (PARP-1) Inhibitor, as an Adjunctive Therapy for the Treatment of Alzheimer’s Disease

**DOI:** 10.3389/fnagi.2020.00255

**Published:** 2020-08-13

**Authors:** Felipe Salech, Daniela P. Ponce, Andrea C. Paula-Lima, Carol D. SanMartin, María I. Behrens

**Affiliations:** ^1^Centro de Investigación Clínica Avanzada, Facultad de Medicina and Hospital Clínico Universidad de Chile, Santiago, Chile; ^2^Sección de Geriatría Hospital Clínico Universidad de Chile, Santiago, Chile; ^3^Departamento de Neurociencia, Facultad de Medicina, Universidad de Chile, Santiago, Chile; ^4^Biomedical Neuroscience Institute, Facultad of Medicina, Universidad de Chile, Santiago, Chile; ^5^Institute for Research in Dental Sciences, Facultad de Odontología, Universidad de Chile, Santiago, Chile; ^6^Center for Integrative Biology, Facultad de Ciencias, Universidad Mayor, Santiago, Chile; ^7^Departamento de Neurologiìa y Neurocirugiìa, Hospital Cliìnico Universidad de Chile, Santiago, Chile; ^8^Departamento de Neurología y Psiquiatría, Clínica Alemana de Santiago, Santiago, Chile

**Keywords:** nicotinamide, PARP-1 inhibitors, Alzheimer’s disease therapy, oxidative death, lymphocytes, parthanatos

## Abstract

Nicotinamide (vitamin B3) is a key component in the cellular production of Nicotinamide Adenine Dinucleotide (NAD) and has long been associated with neuronal development, survival and death. Numerous data suggest that nicotinamide may offer therapeutic benefits in neurodegenerative disorders, including Alzheimer’s Disease (AD). Beyond its effect in NAD^+^ stores, nicotinamide is an inhibitor of Poly [ADP-ribose] polymerase 1 (PARP-1), an enzyme with multiple cellular functions, including regulation of cell death, energy/metabolism and inflammatory response. PARP-1 functions as a DNA repair enzyme but under intense DNA damage depletes the cell of NAD^+^ and ATP and leads to a non-apoptotic type of cell death called Parthanatos, which has been associated with the pathogenesis of neurodegenerative diseases. Moreover, NAD^+^ availability might potentially improve mitochondrial function, which is severely impaired in AD. PARP-1 inhibition may also exert a protective effect against neurodegeneration by its action to diminish neuroinflammation and microglial activation which are also implicated in the pathogenesis of AD. Here we discuss the evidence supporting the use of nicotinamide as adjunctive therapy for the treatment of early stages of AD based on the inhibitory effect of nicotinamide on PARP-1 activity. The data support evaluating nicotinamide as an adjunctive treatment for AD at early stages of the disease not only to increase NAD^+^ stores but as a PARP-1 inhibitor, raising the hypothesis that other PARP-1 inhibitors – drugs that are already approved for breast cancer treatment – might be explored for the treatment of AD.

## Introduction

Aging is the primary risk factor for most neurodegenerative diseases and accordingly several hallmarks of aging, including genomic instability, telomere attrition, epigenetic alterations, loss of proteostasis, mitochondrial dysfunction, cellular senescence, deregulated nutrient sensing, stem cell exhaustion and altered intercellular communication participate in the pathophysiology of neurodegenerative diseases (Hou et al., 2019). Considering these data, several interventions oriented to modulate hallmarks of aging have been proposed for the treatment of neurodegenerative diseases (Hou et al., 2019). In this regard, nicotinamide adenine dinucleotide (NAD) is a universal intracellular electron transporter that plays a central role in most of the processes shared by aging and neurodegeneration (Hou et al., 2019). In cells, NAD can be found in its oxidized form (NAD^+^) and reduced (NADH). In addition to its role in redox metabolism, NAD^+^ is a substrate in several important reactions such as DNA repair via sirtuins (SIRTs), the maintenance of intracellular calcium homeostasis and epigenetically modulated gene expression, with roles in senescence, inflammation and immune reactions (Berger et al., 2004). During the aging process the levels of NAD^+^ decline and agents that elevate intracellular NAD^+^ have shown promising results against neurodegeneration and aging (Verdin, 2015; Braidy et al., 2019; Hou et al., 2019).

Nicotinamide or niacinamide is the water-soluble amide form of vitamin B3 (niacin) with a central role in the synthesis of NAD. Nicotinamide is directly converted to Nicotinamide mononucleotide (NMN) by the enzyme nicotinamide phosphoribosyltransferase (NAMPT) which is then conjugated with ATP and converted to NAD^+^. The rate of NAD^+^ synthesis in this pathway is determined by the conversion of nicotinamide and 5-phosphoribosyl-1-pyrophosphate (PRPP) into NMN by NAMPT in the first step (Hong et al., 2020). Nicotinamide has long been associated with neuronal development, survival, and death. Severe vitamin B3 deficiency is known to cause Pellagra, a clinical condition characterized by diarrhea, skin disorders, and cognitive impairment, the latter suggesting a role for vitamin B3 in maintaining normal brain physiology and in the pathogenesis of diseases associated with cognitive problems. Several lines of evidence suggest that nicotinamide protects neurons from various injuries including trauma, ischemia, and stroke (Mokudai et al., 2000; Vonder Haar et al., 2011). In addition, there is preliminary evidence that it may offer therapeutic benefits in neurodegenerative disorders, such as Alzheimer’s Disease (AD) (Fricker et al., 2018; Hong et al., 2020).

Alzheimer’s Disease is a neurodegenerative disease and the most frequent cause of dementia. It is characterized neuropathologically by the deposit of amyloid-β (Aβ) peptides forming extracellular neuritic plaques and the intracellular accumulation of phosphorylated tau in neurofibrillary tangles. Clinically there is a progressive loss of episodic memory and learning, orientation, judgment, and behavior leading to an important disability and dependence of the elderly and a significant burden to their caregivers (Alzheimer’s Disease International, 2020). The most accepted hypothesis for the pathogenesis of AD proposes that the initial event is the deposit of Aβ, followed by the intracellular accumulation of hyperphosphorylated tau, leading to mitochondrial dysfunction oxidative, synaptic damage and neuronal death associated with inflammation and an immune reaction (Selkoe and Hardy, 2016). However, this hypothesis has been challenged recently mostly due to the failure of anti-amyloid therapies.

There are no curative treatments for AD; the drugs currently approved by the FDA are modulators of the action of two neurotransmitters; anticholinesterases – that facilitate cholinergic transmission – and memantine that regulates glutamatergic transmission, but their effect is modest and only to slow the progression of the disease. Numerous clinical trials have been implemented to test new drugs for the treatment of AD in the last 20 years, mostly based on the amyloid hypothesis probing different anti-amyloid – and also anti tau – drugs. Unfortunately, despite the enormous efforts, both scientific and economic (Cummings et al., 2018), there have been no new FDA approved treatments for AD since 2003, when memantine was approved (Reisberg et al., 2003). Therefore, there is an urgent need for new therapeutic options for the treatment of AD.

### Cell Death and AD

In addition to amyloid plaques and neurofibrillary tangles, AD is characterized by extensive neuritic and synaptic degeneration and neuronal cell death. The type of cell death in AD is still controversial, but it is clear there is progressive atrophy of the brain due to cell and synaptic loss. Notably, brain atrophy starts in asymptomatic individuals several years before the onset of dementia, however, the precise mechanism by which neurons die remains unknown (Chen and Mobley, 2019). Classically, cell death has been classified into two main groups: apoptosis and necrosis. Apoptosis is a programmed form of cell death, whereas necrosis is an uncontrolled lysis of the cell. However, more recently there has been growing appreciation for other cell death mechanism (Jellinger, 2001) including necroptosis and parthanatos, both programmed form of necrosis (Mandir et al., 1999; Wang et al., 2009; Caccamo et al., 2017). Parthanatos is a form of caspase-independent cell death that shares characteristics of apoptosis and necrosis. It is activated under conditions of severe cell injury, like intense DNA oxidative/nitrosative damage that activates Poly [ADP-ribose] polymerase 1 (PARP-1) (Wang et al., 2011; Fatokun et al., 2014; Dawson and Dawson, 2017). PARP-1 hyperactivation consumes large amounts of cellular NAD^+^ and ATP, which in turn activates the release of apoptosis inducing factor (AIF) from mitochondria that translocates to the nucleus, producing chromatin condensation and largescale DNA fragmentation (Wang et al., 2011). The activated DNA repair mechanism are overwhelmed leading to a massive consumption of NAD^+^ and ATP, resulting in cell death, which is no longer by apoptosis, since apoptosis requires ATP (Fatokun et al., 2014; Dawson and Dawson, 2017). Notably there are reports indicating that parthanatos can occur without changes in AIF (Jang et al., 2017), and the increase in PARP activity remains the hallmark of this type of death (Salech et al., 2017). Interestingly, nicotinamide is a well-known inhibitor of PARP-1.

### Mitochondria and AD

More recently different lines of evidence support a role for mitochondrial dysfunction as a central event in the pathogenesis of AD, where several mitochondrial parameters such as oxygen consumption, ATP production, ROS generation, Ca^2 +^ signals, mitochondrial dynamic and mitophagy, are altered (Martire et al., 2015; Guo et al., 2017; Sanmartín et al., 2017; Swerdlow, 2018; Flannery and Trushina, 2019; Huang et al., 2019). Electron transfer chain enzyme activities in autopsy AD-brains show disturbances in all mitochondrial complexes, most marked in complex IV cytochrome c oxidase (COX) (Maurer et al., 2000). Alterations in synaptic mitochondria have also been demonstrated in a transgenic AD-mouse model (Du et al., 2010). Major subunits of F1FO-ATP synthase are selectively decreased in Mild Cognitive Impairment (MCI) and AD human brains and 5×FAD mice, affecting ATP production and the maintenance of the mitochondrial membrane potential (Δψ) (Beck et al., 2016). In hippocampal neuronal cultures Aβ induces neuronal apoptosis by targeting mitochondria, prompting disruption of mitochondrial membrane potential, increasing reactive oxygen species (ROS) generation and activating the process of mitophagy (Yang et al., 2009; Han et al., 2017). Disruption of mitochondrial dynamics induces functional disorders in the mitochondrial network, such as failed energy production, deregulation in Ca^2 +^ homeostasis and generation of ROS, which are closely related to the physio-pathological changes associated with AD (Wang et al., 2014; Kim et al., 2017). Soluble oligomers of Aβ induce abnormal low-intensity and long-lasting Ca^2 +^ signals in the cytoplasm and an increase in the content of Ca^2 +^ in the mitochondria, which is followed by increased in mitochondrial superoxide and hydrogen peroxide (H_2_O_2_) production in rat hippocampal neurons, causing fragmentation of the mitochondrial network (Paula-Lima et al., 2011; SanMartín et al., 2014; Sanmartín et al., 2017). Of note, pre-incubation with the antioxidant agent N-acetyl cysteine (NAC), a physiological precursor of cellular glutathione, or with EUK-134, a mitochondrial antioxidant agent, prevented mitochondrial ROS production and mitochondrial fission, and the spatial memory deficits in a rat model of AD (Sanmartín et al., 2017; More et al., 2018). Moreover, in fibroblasts from AD patients we demonstrated a reduction in mitochondrial length associated with changes in the regulation of a fusion-regulating protein and opening of the mitochondrial transition pore, compared to fibroblasts from aged-matched controls and young subjects (Pérez et al., 2017, 2018).

In all these examples, mitochondrial dysfunction is secondary to the direct or indirect effects of Aβ on mitochondria. Alternatively, there is evidence to support a primary mitochondrial dysfunction, known as the “mitochondrial cascade theory” (Swerdlow, 2018). According to this hypothesis, impaired mitochondrial function and the associated bioenergetic changes alter Aβ homeostasis and lead to the accumulation of Aβ. For example, it is well known that mitochondria-generated ROS play a role in shifting APP processing to generate Aβ (Leuner et al., 2012). Besides, several cell culture experiments suggest that interfering cell bioenergetics shifts APP processing toward the amyloidogenic pathway (Gasparini et al., 1997; Webster et al., 1998). Furthermore, studies in non-brain tissues, such as platelets, fibroblast, muscle or lymphocytes from AD patients, where neurodegeneration *per se* should not directly cause specific biochemical defects, have documented mitochondrial disfunction, supporting the hypothesis of a primary mitochondrial disfunction in AD. It is also feasible that both primary and secondary mitochondrial cascades co-exist in AD, aggravating the condition in a progressive way (Swerdlow, 2018).

### Inflammation and AD

A role for inflammation in AD has been debated for a long time and it is still uncertain whether inflammation is a cause, contributor, or secondary phenomenon in the disorder (Wyss-Coray and Rogers, 2012; Heneka et al., 2015; Schwartz, 2017). The original description of Alois Alzheimer in 1905 (Stelzmann et al., 1995) indicates the presence of activated microglia –the main innate immunity cell in the central nervous system. However, they were considered merely an epiphenomenon of late stages of the disease. Conversely, nowadays it is accepted that an inflammatory and immune reaction of the innate and adaptive immune system are key factors in the pathogenesis of AD (Heneka et al., 2015; Marsh et al., 2016; Jevtic et al., 2017; Prinz and Priller, 2017). Neuropathological data show the presence of microglial cells and reactive astrocytes in the vicinity of the Aβ deposits (Verkhratsky et al., 2016) and reactive glia are responsible for the associated neuroinflammation and the secondary glial and neuronal dysfunction present at certain stages of AD (Hemonnot et al., 2019). It is proposed that the constant presence of Aβ would turn microglia “exhausted” and ineffective in the long run (McGeer and McGeer, 1998; Heneka et al., 2014; Heppner et al., 2015; Navarro et al., 2018). In support of inflammation playing an important role in AD, several polymorphisms in key microglial genes encoding factors involved in phagocytosis and clearance of misfolded proteins are associated with increased risk of AD (Bradshaw et al., 2013; Guerreiro et al., 2013; Jonsson et al., 2013). Schwartz and cols proposed the interesting hypothesis that brain aging and neurodegenerative diseases are associated with a dysfunction of the blood-CSF barrier that impedes the entrance of protective peripheral immune cells (Baruch et al., 2014, 2015) and that releasing the brake of the immune system may be beneficial for AD (Baruch et al., 2015, 2016).

Retrospective and observational studies show a beneficial association between the long-term use of non-steroidal anti-inflammatory drugs and the risk of AD (in ’t Veld et al., 2001), but newer large-scale clinical trials have not corroborated these results (Aisen et al., 2000, 2003). This contradiction might be due to the timing and/or duration of the anti-inflammatory treatment. For example, non-steroidal anti-inflammatory drugs have a beneficial role when administered preclinically for 2–3 years or at ages younger than 65, but an adverse effect at later stages of AD pathogenesis (Breitner et al., 2011; Tschanz et al., 2013), suggesting a therapeutic window for the use of NSAIDS. However, a recent clinical trial of NSAIDS was negative (Meyer et al., 2019) and currently, anti-inflammatory drugs are not recommended for the treatment of AD.

### PARP-1 and Its Role in Cell Death, Mitochondrial Function, and Inflammation in AD

There is increasing evidence of the involvement of PARP-1 in neurodegeneration (Cosi et al., 2004; Narne et al., 2017). PARP-1 belongs to a family of enzymes that transfer ADP-ribose moieties from NAD^+^ to a variety of acceptor proteins, among them PARP-1 itself, constructing a net of ADP-ribose polymers, a process called “PARylation.” The most known function of PARP-1 is nuclear DNA repair (Pascal, 2018). However, the variety of proteins that are parylated by PARP-1 makes this enzyme an important factor in diverse cellular processes. As mentioned above, in settings of intense oxidative DNA damage, the strong activation of PARP-1 and formation of PAR polymers consumes large amounts of cellular NAD^+^ and ATP, which leads to Parthanatos (Fatokun et al., 2014; Dawson and Dawson, 2017). This type of death, is observed in different cells of the organism, including neurons (Lee et al., 2013), and is present in neurodegenerative disorders such as Parkinson’s disease and AD (Langston et al., 1983; Fatokun et al., 2014). Increased Parylation of nuclear proteins was reported in AD human brains (Love et al., 1999). In transgenic mice AD brains PARP-1 activation is present at early stages of amyloid deposit (Martire et al., 2013). Furthermore, crossing of an AD transgenic mouse with a PARP-1 (−/−) mouse was able to prevent cognitive dysfunction, synaptic damage and microglial activation (Kauppinen et al., 2011). Also, the injection of Aβ into PARP-1 (−/−) mice or the inhibition of PARP-1 with PJ34 induced lower microglial activation compared to controls (Kauppinen et al., 2011). Recent studies show that the toxicity of Aβ involves the activation of the transient receptor potential melastatin-related 2 (TRPM2) channel, a cation channel activated by ADP-ribose (Miller, 2006; Alawieyah Syed Mortadza et al., 2018; Li and Jiang, 2018; Raghunatha et al., 2020). In studies in hippocampal neurons it is proposed that Aβ-generated ROS damage the DNA activating PARP-1 which in turn activates TRPM2 leading to Ca^2+^ entry and mitochondrial dysfunction perpetuating the cycle (Li and Jiang, 2018). The KO of TRPM2 suppressed microglial activation in the APP/PS1 transgenic AD mouse model (Ostapchenko et al., 2015) and involvement of astrocytic PARP-1 is also described by Aβ damage (Abeti and Duchen, 2012; Angelova and Abramov, 2014). Furthermore, recent evidence shows a reduced level of PARP-1 nucleolar immunohistochemical staining in hippocampal pyramidal cells in AD and MCI patients (Regier et al., 2019). These findings strongly support a key role for PARP-1 in Parthanatos, mitochondrial function and inflammation occurring in AD.

### Protective Effects of Nicotinamide as a Parp-1 Inhibitor

**FIGURE 1:**
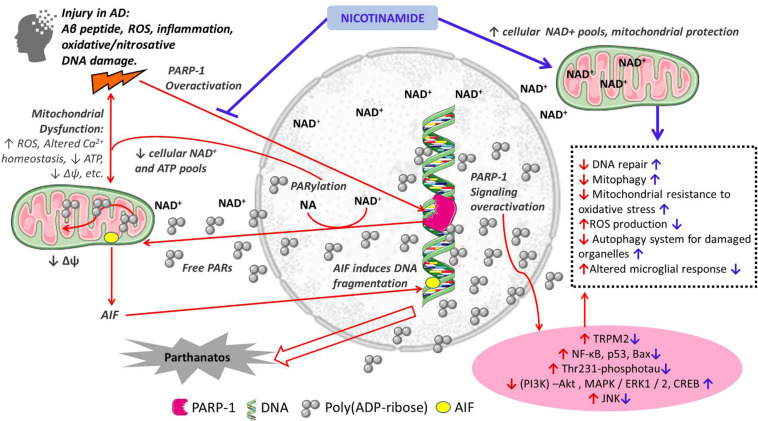
Schematic representation of the proposed mechanisms of action of nicotinamide in the pathogenesis of Alzheimer’s disease. Red arrows indicate mechanisms of cellular damage in AD: pro-death, mitochondrial dysfunction and inflammation; Blue arrows indicate protective effects of nicotinamide directly as a substrate for NAD^+^ synthesis with its effects on cellular metabolism, and through PARP-1 inhibition and its beneficial effects preventing cell death, mitochondrial dysfunction and inflammation. Other PARP-1 inhibitors may have similar effects.

Nicotinamide as a precursor of NAD^+^ has numerous important functions in cellular metabolism. Therefore, the supplementation of nicotinamide is relevant to the maintenance of NAD^+^ pools and their role as a cofactor of sirtuins, DNA repair, mitochondrial health and Ca^+^ homeostasis, autophagy of damaged cells all important in AD pathogenesis (Figure 1). In the following sections we discuss the potential additional role of nicotinamide as a PARP-1 inhibitor in the pathogenesis of AD through its role in cell death, mitochondrial function and inflammation.

### PARP-1 and Cell Death

The tight connection between nicotinamide as a precursor of NAD^+^ is important in the regulation of cell death. The protective role of nicotinamide in neurodegenerative disorders might be associated with the function of nicotinamide as a PARP-1 inhibitor. In studies with human lymphocytes we reported that exposure to H_2_O_2_ induces a caspase-independent death that is strongly protected by PARP-1 inhibition with 3-aminobenzamide (3-ABA), suggesting it corresponds to Parthanatos (Behrens et al., 2011). Furthermore, this oxidative death was age-dependent; healthy young subjects showed predominantly necrotic death whereas apoptosis prevailed in older individuals as evidenced by flow cytometry, electron microscopy and caspase activity (Behrens et al., 2011). Moreover, we demonstrated that lymphocytes from cognitively impaired patients are more susceptible to H_2_O_2_-induced death which correlated with the degree of dementia severity (Behrens et al., 2012; Ponce et al., 2014). The death was markedly protected by PARP-1 inhibition with 3-ABA and also by nicotinamide (Ponce et al., 2014; Salech et al., 2017). Intriguingly, the protection was complete in MCI, but not AD lymphocytes (Salech et al., 2017), suggesting that PARP-1 inhibition might be useful at initial stages of the disease, consistent with the existence of a therapeutic window to protect from cell death at early stages of neurodegeneration, before the damage is too widespread (Salech et al., 2018).

### PARP-1 and Mitochondrial Dysfunction

The presence of PARP-1 in mitochondria and its role in mitochondrial function is still a matter of debate (Brunyanszki et al., 2016; Kadam et al., 2020), which gives novelty and interest to the topic. Parylated proteins are abundant in mitochondria (Bai et al., 2015) and overactivation of PARP consumes mitochondrial NAD^+^. Mitochondria contain a significant proportion of the cellular NAD^+^ (Alano et al., 2007) and the excessive consumption of NAD^+^ in relation to cytotoxic damage modifies the activity of mitochondrial energetic synthesis, affecting the production of ROS. Beyond, PARP-1 inhibits mitochondrial respiration in neurons by suppressing the mitochondrial regulator peroxisome proliferator-activated receptor γ coactivator-1α (PGC-1α) (Lu et al., 2019). Furthermore, there is more controversial evidence showing that PARP-1 could exert a direct effect on mitochondria including parylation of mitochondrial proteins and mitochondrial DNA repair, processes mediated by mitofilin, a molecule that acts as a dock for PARP-1 mitochondrial activity, since PARP-1 lacks a classical mitochondrial localization signal (Rossi et al., 2009). Contrary to its action in the nucleus, in mitochondria PARP-1 is a negative regulator of DNA repair (Szczesny et al., 2014). Nevertheless, even though the role of mitochondrial PARP-1 is unclear, there is evidence of beneficial effects of PARP-1 inhibition on mitochondrial and glycolytic functions induced by oxidative stress (Martire et al., 2016; Gallyas and Sumegi, 2020). Interestingly, the most vulnerable cells to PARP-1 overactivation are those that rely on aerobic glycolysis, such as the default mode network, since NAD^+^ and ATP depletion is preferentially observed in the cytosol and nucleus and not inside mitochondria (Kim et al., 2017). This is intriguing, since the default mode network is where Aβ is initially deposited in AD brains (Vlassenko et al., 2010).

### PARP-1 in Inflammation and Microglial Activation

Beyond the roles of PARP-1 in DNA repair and cell death, PARP-1 is involved in inflammation by regulating the expression of pro-inflammatory factors. Poly-(ADP)-ribosylation of histones results in the transcription of pro-inflammatory genes, such as several NF-κB-dependent cytokines, chemokines, adhesion molecules, and inducible nitric oxide synthase (*iNOS*) (Sethi et al., 2017). Interestingly, *in vitro* studies show that the response of microglial to inflammation is mediated by PARP-1. Studies in microglial cultures from PARP-1 knock-out mice demonstrate that PARP-1 is necessary for the activation of microglia upon TNF-α stimulation (Kauppinen and Swanson, 2005). In a microglial cell line (BV2 cells) microglial activation induced by lipopolysaccharides (LPS) stimulation prompted PARP-1 enzymatic activity that enhanced microglial expression of NF-κB-dependent inflammatory cytokines (IL1β and TNF-α) (Martínez-Zamudio and Ha, 2014). The signaling pathway maintaining microglial PARP−1 activation also involves the TRMP2 channel (Raghunatha et al., 2020). *In vivo* experiments are in line with this evidence. A mouse model of traumatic brain injury showed that systemic administration of PJ34, a selective inhibitor of PARP-1, reduced microglial activation in the brain cortex (Stoica et al., 2014). As mentioned above, PARP-1 inhibition or KO in transgenic AD mice had a beneficial effect on cognitive dysfunction, synaptic damage and microglial activation (Kauppinen et al., 2011).

### Nicotinamide Treatment in Animal Models of Neurodegeneration

Treatment with different derivatives of niacin: nicotinamide, nicotinamide mononucleotide (NMN), or nicotinamide riboside, a precursor of neuronal NAD^+^ synthesis have all shown beneficial effects in mouse models of AD. In the transgenic 3×Tg-AD mouse model, Green and collaborators demonstrated that nicotinamide treatment restored cognitive deficits through a reduction of phosphorylated species of tau (Thr231-phosphotau) (Green et al., 2008). This effect on Thr231-phosphotau was also regulated by Sirtuin1, a NAD+ depending histone deacetylase (HDAC), supporting the hypothesis that nicotinamide can act as a HDAC inhibitor (Green et al., 2008). In the same animal model, Liu and collaborators reported that administration of nicotinamide resulted in improved cognitive performance and decreased Aβ and hyperphosphorylated tau deposits in the hippocampus and cerebral cortex (Liu et al., 2013). Besides, they showed that nicotinamide treatment activated signaling pathways critical for neuronal survival and synaptic plasticity including Akt, extracellular signal-regulated kinases (ERKs), and the transcription factor cyclic AMP response element-binding protein (CREB). The authors suggest that nicotinamide treatment improved brain bioenergetics and preserved the functionality of mitochondria and the autophagy system (Liu et al., 2013). In another transgenic mouse model of AD, the APPswe/PS1dE9 (AD-Tg) mouse, the administration of NMN lead to significantly decreased β-amyloid production, amyloid plaque burden, synaptic loss, and inflammatory responses through JNK activation, which was accompanied by a substantial improvement in behavioral measures of cognitive impairments compared to control AD-Tg mice (Yao et al., 2017). Non-transgenic animal models of AD are also available for the study of AD. Bilateral stereotaxic injection of Aβ(1–42) oligomers into the hippocampus of Sprague–Dawley rats induces memory deficits and histopathological alterations in the brains of animals (More et al., 2018; Concha-Miranda et al., 2020). In this animal model the administration of intraperitoneal nicotinamide for 7 days was associated with downregulation of the elevated levels of PARP-1, as well as of NF-κB, p53, Bax and oxidative stress parameters (Turunc Bayrakdar et al., 2014). In another study, treatment with intraperitoneal NMN was accompanied by sustained improvement in cognition as assessed by the Morris water maze in rats infused with Aβ oligomers in the cerebral ventricles (Wang et al., 2016). In addition, in organotypic hippocampal slice cultures (OHCs) treated with Aβ oligomers, the authors demonstrated that NMN attenuated neuronal cell death, prevented the inhibition of long-term potentiation (LTP), restored the levels of NAD^+^ and ATP and eliminated the accumulation of reactive oxygen species (Wang et al., 2016). A protective effect of nicotinamide has also been demonstrated in another neurodegenerative disorder; in a rat model of Huntington’s disease induced by the administration of 3-nitropropionic acid (3-NP), the administration of nicotinamide 100, 300, and 500 mg/Kg intraperitoneally protected from the motor detrimental effects of 3-NP that was accompanied by decreased oxidative stress markers (malondialdehyde, nitrites) and increased antioxidant enzyme (glutathione) levels (Sidhu et al., 2018). In all, these results suggest an *in vivo* neuroprotective role of nicotinamide in different animal models of AD and Huntington’s disease. The mechanism by which nicotinamide exerts its protective effect in animal models of neurodegenerative is unknown.

## Discussion

The protective effects of nicotinamide and PARP-1 inhibition on oxidative death, mitochondrial dysfunction and microglial activation, in addition to the well-known beneficial effect of increasing NAD^+^ pools support the idea that nicotinamide and other PARP-1 inhibitors constitute a new pathway to find molecular targets for the treatment of early stages of AD (Figure 1). The evidence discussed supports the use of nicotinamide as an adjunctive and simple treatment to prevent oxidative cell death, mitochondrial dysfunction and deleterious microglial inflammation at early stages of AD (Figure 1). It is interesting to note that a Double-Blind-Randomized, Placebo-Controlled Trial has been launched to study the role of Nicotinamide in Mild Cognitive Impairment or mild Alzheimer’s disease (ClinicalTrial ID NCT03061474^1^). The purpose of this research is to test whether high dose nicotinamide (750 mg tablets taken orally twice daily) can reduce the levels of phosphorylated tau assessed in the cerebrospinal fluid. However, the goal of this trial is not the evaluation of nicotinamide as a PARP-1 inhibitor.

Nicotinamide can freely cross the blood-brain barrier in both directions (Spector, 1987) and has a well-described safety profile. Besides, it is widely available commercially in many countries as 500 mg tablets, and also at doses of 14–50 mg in a range of multivitamin preparations. The actions of PARP-1 are not confined to the brain; it is known to participate in peripheral mechanisms in response to oxidative stress and inflammatory molecular signaling. Moreover, there are suggestions that AD might be a systemic disorder (Morris et al., 2014; Van Der Velpen et al., 2019). Peripheral lymphocytes, fibroblasts and plasma of AD patients show alterations of the expression and function of the cellular machinery that correlate with the pathology present in the brain (Ponce et al., 2014; Saresella et al., 2016; Pérez et al., 2017). Therefore, PARP-one inhibition and increases in NAD^+^ pools might also be beneficial for AD by constraining processes of systemic damage that may be indirectly contributing to brain pathology.

The utility of a treatment with nicotinamide or other PARP-1 for AD might be more effective at early stages of the disease, before overt neurodegeneration and the deleterious effects of mitochondrial and exhausted microglial dysfunction are widespread. In a study in cultured microglia, those extracted from adult animals showed diminished capacity to phagocytose Aβ fibrils compared with microglia from postnatal animals (Floden and Combs, 2011). It is also interesting to mention, that PARP-1 inhibition might have different effects depending on cell type, for example, PARP-1 overactivation lead to cell death in neurons, however, in microglia it induced protein synthesis and proliferation (Kauppinen and Swanson, 2005; Kauppinen et al., 2011). This suggests there might be an optimal timing for the use of PARP-1 inhibition in the treatment of AD.

Several PARP-1 inhibitors have been developed for cancer therapy and are currently available, including olaparib, niraparib, rucaparib, veliparib, and talazoparib (McCann and Hurvitz, 2018). Eventually, these inhibitors could be explored for early AD treatment. Although the safety issue in long-term administration of the existing PARP-1 inhibitors remains open, their potential as a possible adjunctive treatment for AD should be further studied. In summary, there are a series of experimental and clinical studies supporting the role of PARP-1 inhibition – with nicotinamide or other PARP-1 inhibitors – as a potential adjunctive therapeutic target for AD that deserves further research in this field. The ongoing clinical trial of nicotinamide is testing its role as a HDAC inhibitor on tau phosphorylation. Our hypothesis adds to the field to include PARP-1 inhibition in future directions including clinical trials to evaluate neuronal injury.

## Author Contributions

FS contributed with the general idea, discussion, and elaboration of the first manuscript draft, and revision of the final manuscript. DP, AP-L, and CS contributed with the general idea, discussions, and revision of the final manuscript. MB contributed with the general idea, discussions, writing and final revision of the manuscript, and general direction. All authors contributed to the article and approved the submitted version.

## Conflict of Interest

The authors declare that the research was conducted in the absence of any commercial or financial relationships that could be construed as a potential conflict of interest.

## Footnotes

1 https://clinicaltrials.gov/ct2/show/NCT03061474
